# Multivariate Single-Step GWAS Reveals Pleiotropic Genomic Regions and Candidate Genes Associated with Male Scrotal Circumference and Female Fertility Traits in Retinta Beef Cattle

**DOI:** 10.3390/vetsci12100977

**Published:** 2025-10-11

**Authors:** Chiraz Ziadi, Rosa María Morales, María Ángeles Vargas-Pérez, Gabriel Anaya Calvo-Rubio, Sebastián Demyda-Peyrás, Antonio Molina

**Affiliations:** Department of Genetics, University of Córdoba, 14071 Córdoba, Spain; z72zizic@uco.es (C.Z.); v22mocir@uco.es (R.M.M.); z42vapem@uco.es (M.Á.V.-P.); b22ancag@uco.es (G.A.C.-R.); ge2depes@uco.es (S.D.-P.)

**Keywords:** scrotal circumference, female fertility traits, ssGBLUP, GWAS, Retinta beef cattle

## Abstract

**Simple Summary:**

Reproduction plays a crucial role in the long-term sustainability of livestock, particularly in extensive production systems. However, reproductive traits typically exhibit low heritability, mainly due to the general lack of objective performance control criteria related to fertility in breeding females, and the fact that these traits are subject to numerous environmental effects that are difficult to quantify. This means that, in many beef cattle breeds, an indirect selection criterion is being used, such as the male’s scrotal circumference, because it is considered to be associated with the fertility of his daughters. This would imply the existence of genes with pleiotropic effects in both sexes. To better understand the genetic architecture underlying these traits, a genome-wide association study was conducted for scrotal circumference (SC) and several female fertility traits within the Retinta cattle breed. Genes previously reported in cattle breeds for SC and female fertility traits were identified in this study. Two of these main genes were detected in the GWAS of the bull’s scrotal circumference and parameters related to cow fertility.

**Abstract:**

Fertility is key for calf production. Direct selection for female fertility under field conditions is hindered by low accuracy and selection response. An alternative widely implemented is selection for scrotal circumference (SC), genetically correlated with daughter fertility. This study performed a genome-wide association study (GWAS) to identify genomic regions and candidate loci linked to SC and female fertility in Retinta cattle. A multivariate ssGBLUP was applied using SC records from 1061 bulls, fertility-related traits from 59,254 females and genotypes from 1230 animals using the Axiom™ Bovine Genotyping v3 Array (65k). The ssGWAS revealed 23 1-Mb windows explaining >1% of additive genetic variance for SC, one on chromosome 2 and 22 on chromosome 3. Within these windows, 198 regions spanning 118 protein-coding genes and 80 RNA genes were identified. Several genes, including *GSTM3*, *SPATA1*, *HFM1*, and *MSH4*, were previously associated with male fertility. Six regions overlapped across male and female traits, containing two protein-coding genes (*THSD7B* and ENSBTAG00000021755). Identification of genomic markers linked to both female fertility and male SC enables selection of superior animals, improving reproductive efficiency and advancing knowledge of the genomic basis of male–female fertility relationships.

## 1. Introduction

Fertility is a key economic trait in beef cattle production systems, as reproductive efficiency directly impacts overall productivity and profitability [1,2]. Enhancing reproductive performance is, therefore, of major economic importance, with total lifetime productivity, determined by both reproductive success and output per cow, serving as a critical metric of efficiency [3,4]. However, assessing female reproductive performance objectively presents significant challenges due to the variability of environmental and management conditions, compounded by the generally low associated heritability [5,6]. Additional challenges are related to the collection of records from extensively managed herds and the limited availability of reliable data for genetic evaluations [7]. Consequently, selection programs often rely on indirect selection criteria, with scrotal circumference being one of the most widely applied. Scrotal circumference (SC) in bulls is relatively easy to measure and exhibits moderate to high heritability [6,8,9,10]. Furthermore, this trait has been shown, through quantitative studies, to be genetically correlated with the reproductive traits of female offspring, supporting the feasibility of improving beef cattle fertility by selecting to increase SC in sires [11,12,13]. Bulls with larger SC tend to reach puberty earlier, transmit reproductive precocity to their offspring, and produce daughters with longer productive lifespans [14,15].

The Retinta is an autochthonous Spanish cattle breed, predominantly raised in the southern and western regions of Spain, where around 90% of the national population is concentrated. Nonetheless, the breed is also present in other countries, including Portugal, Argentina, and Brazil [16]. The National Association of Retinto Breeders (ACRE) estimates the population at 250,000 cows, of which 20,715 animals belonged to the performance control nucleus and the breed improvement program in 2023, as reported by the Spanish Ministry of Agriculture, Fisheries, and Food [17].

The Retinta is reared under an extensive management system within a distinctive habitat known as the dehesa, a unique Mediterranean ecosystem characterized by a combination of mountain shrublands and open oak woodlands, featuring diverse tree and shrub species [18]. This breed is well adapted to semi-arid, dry, and hot environments with limited resources [19], and is noted for its strong maternal abilities [20].

Routine genetic evaluations in the Retinta breed have traditionally been based on the BLUP (Best Linear Unbiased Prediction) methodology, using only phenotypic performance and pedigree information, with a focus on traits such as growth and longevity [16,20]. In recent years, female fertility has been introduced as a selection objective, using reproductive efficiency as the selection criterion [21]. For years, scrotal circumference in bulls has been used as an indirect selection criterion to improve cow fertility [10,22].

In recent years, however, genotyping has been introduced, paving the way for genomic evaluations of economically important traits. In this context, the single-step genomic best linear unbiased prediction (ssGBLUP) has emerged as the reference method for genomic evaluations [23,24]. This methodological approach enables the integration of all available data from both genotyped and non-genotyped animals, namely phenotypic, pedigree, and genomic data into the same framework [25].

It is important to highlight that the development of high-density genotyping panels in genome-wide association studies (GWAS) enables the precise identification of genomic regions, candidate genes, and specific markers linked to economically important traits. This enhances understanding of the genetic mechanisms underlying these traits and supports the development of more effective animal breeding programs [26]. Among these traits, fertility has received growing attention, as reproductive efficiency is crucial for livestock productivity and sustainability. DNA-based technologies and the emergence of genomics have led to significant advances in identifying genetic variants affecting fertility [27]. Genome-wide association studies (GWAS) conducted on beef cattle breeds have revealed several potential genes for female fertility traits, including age at first calving [28,29], calving interval [30], pregnancy status [31], gestation length [32], sexual precocity [33], and bull scrotal circumference [9]. Moreover, other studies that combined female fertility traits with male body weight at puberty [34] or with SC [35,36] identified common genomic regions harboring genetic variation that may influence reproductive traits in both sexes. In the Retinta breed, we recently described multiple genes associated with reproductive efficiency [21], as well as several female-related fertility traits [37]. However, no studies are available that relate scrotal circumference to female fertility traits in Retinta cattle.

Therefore, the present study aimed to estimate genetic parameters and correlations between scrotal circumference (SC) and several cow fertility-related traits, and to conduct a GWAS for these traits within the Retinta cattle breed to better understand the genetic architecture underlying the SC traits and the relationship between SC and fertility traits.

## 2. Materials and Methods

### 2.1. Ethical Statement

The samples used in this study were obtained during compulsory sanitary practices by official veterinarians, avoiding disturbing the animals solely for research purposes. Data were obtained from the official records of the National Association of Breeders of Selected Retinta Cattle (ACRE).

### 2.2. Phenotypic Data and Pedigree

The dataset and pedigree employed in this study were provided by the National Association of Breeders of Selected Retinta Cattle (ACRE). The data for the scrotal circumference (SC) evaluation in the Retinta breed were collected at breeding bull test stations (CENSYRA, Badajoz and CEAG, Cádiz, Spain). The database comprised 1061 SC records collected in test stations with young bulls (8–14 months). The SC was measured monthly, using a measuring tape in cm, to obtain measurement before and after the bull turned one-year-old. These two measurements were used to calculate the standard measurement at 12 months of age. A total number of 1061 young bulls were measured for SC, originating from 912 dams and 367 sires.

The fertility of the cows was defined using the following traits: age at first calving (AFC) of the whole population, interval between first and second calving (CI12), average calving interval (ACI), and reproductive efficiency at last calving (RE), which was calculated as the percentage deviation between the optimal and real parity number of females at each age, following the procedure described by Jiménez et al. [21].

The dataset for female traits comprised calving records from 69,212 cows that produced offspring with 3630 different bulls. After data editing, 59,254 cows were retained for genetic analysis. To obtain the genealogical kinship matrix, the pedigree was extended to include all the available information in the breed herdbook, with a total of 65,635 animals, including eight complete generations and eleven equivalent generations of ancestors.

### 2.3. Genotyping and Quality Control

A total of 1230 animals (890 bulls and 340 cows) from the whole Retinta population were selected for the genomic assay based on the following criteria: representation of the highest number of herds (88), low level of kinship, completeness of pedigree and data for the studied parameters. Blood samples were collected using EDTA-K3 BD vacutainers™ (BD, Madrid, Spain) by the official technicians of ACRE.

Genomic DNA was isolated from blood using the commercial DNA purification kit DNeasy Blood & Tissue Kit (Qiagen, Germantown, MD, USA), following the manufacturer’s protocol. The quantity and quality of the DNA were measured with a Thermo Scientific™ NanoDrop™ One (Thermo Fisher Scientific Inc., Waltham, MA, USA). Those samples with optimal ratios (absorbance ratios of A260/A280 and A260/230 of 1.8 to 2) were genotyped using the Axiom™ Bovine Genotyping v3 Array (Thermo Fisher Scientific Inc., Waltham, MA, USA), which includes more than 63,000 SNPs. Raw data were processed in the Axiom analysis suite package v5.0 [38] where all SNPs had the highest quality levels of genotyping (DQC ≥ 0.82 and individual call rate QC ≥ 0.90). Subsequently, the dataset was pruned to keep markers from autosomal chromosomes (BTA1 to BTA29). After that, SNPs without correct annotations, SNPs with more than 5% missing genotypes, and those with a minor allele frequency < 0.01 were removed, which left 45,331 variants, using PLINK software v1.9. [39].

### 2.4. Single-Step GBLUP Analysis

The significance of the fixed effects for bull scrotal circumference and cow fertility traits was determined using the ‘GLM2’ package [40] in the R statistical environment v4.4.2 [41]. All fixed effects were significant at the 0.05 level. The genetic evaluation of SC, AFC, CI12, ACI, and RE was performed using a multivariate animal model.

For SC, the following model was fitted:

$$y_{ijkl}=\mu +COV\left(MA\right)+X{ADG}_{i}+Za_{j}+W{cg}_{k}+e_{ijkl}$$

where $y_{ijkl}$ is the dependent variable SC; μ is the overall mean; MA is the mother’s age at the male’s birth as a covariate; ${ADG}_{i}$ is the fixed effect of average daily gain (kg/day) in the test station (3 classes: 1 ≥ 0.62 and <1.10; 2 ≥ 1.10 and <1.30; 3 ≥ 1.30 and ≤1.84); $a_{j}$ is the random additive genetic effect; ${cg}_{k}$ is the random contemporary group of bulls at the test station (99 classes resulting from the combination of start date and test station center); $e_{ijkl}$ is the residual effect; and X, Z, and W are incidence matrices connecting fixed and random effects.

The model assumed for AFC was

$$y_{ijk}=\mu +Za_{i}+W{cg}_{j}+e_{ijk}$$

where $y_{ijk}$ is the dependent variable AFC; µ is the overall mean; $a_{i}$ is the random additive genetic effect; ${cg}_{j}$ is the random contemporary group of cows (12,555 classes resulting from the combination of herd-year-season of birth of the cow); $e_{ijk}$ is the vector of random residuals; and Z and W are incidence matrices relating observations to additive genetic and contemporary group effects, respectively.

Finally, CI12, ACI, and RE were analyzed using the following model:

$$y_{ijkl}=\mu +COV\left(Fc\right)+COV\left({Age}_{l}\right)+X{Age}_{i}+Za_{j}+W{cg}_{k}+e_{ijkl}$$

where $y_{ijkl}$ is the observation of the corresponding trait; µ is the overall mean; Fc is the inbreeding value of the cow, included as a covariate (except for ACI); ${Age}_{l}$ is the age at last calving for RE, included as a covariate; ${Age}_{i}$ is the fixed effect of the age at first calving (3 classes: 1 < 30 months; 2 ≥ 30 and <36; 3 ≥ 36); $a_{j}$ is the random additive genetic effect; ${cg}_{k}$ is the random contemporary group of cows (16,124 classes resulting from the combination of herd-year-season of birth of the cow); $e_{ijkl}$ is the vector of random residuals; and X, Z, and W are incidence matrices relating observations to fixed, additive genetic and contemporary group effects, respectively. The residual covariance was set to zero between scrotal circumference and female reproductive traits, as these traits were measured in different animals.

It was assumed that $a\sim N(0,H\sigma_{a}^{2}$), CG$\sim N(0,I\sigma_{cg}^{2}$), and $e\sim N\left(0,I\sigma_{e}^{2}\right)$ for all traits, where $\sigma_{a}^{2}$, $\sigma_{cg}^{2},$ and $\sigma_{e}^{2}$ are the additive genetic, contemporary group, and residual variances, respectively. The matrix H was obtained following Aguilar et al. [42] by combining the numerator relationship matrix (A) with the genomic relationship matrix (G). The inverse of the H matrix is

$$H^{-1}=A^{-1}+\left[\begin{array}{cc} 0 & 0\\ 0 & G^{-1}-A_{22}^{-1} \end{array}\right]$$

where A is the pedigree-based relationship matrix for all animals; $A_{22}$ is the pedigree-based relationship matrix for genotyped animals; and G is the genomic relationship matrix for genotyped animals, obtained following VanRaden [43] as

$$G=\frac{ZZ’}{\sum_{i=1}^{N}2p_{i}\left(1-p_{i}\right)}$$

where Z is the matrix of SNP genotypes, N is the number of SNPs, and p_i_ is the minor allele frequency of the i-th SNP.

Variance components and genomic estimated breeding values (GEBVs) for the studied traits were estimated using the REML (restricted maximum likelihood) approach with BLUPF90+ software v2.48 [44].

### 2.5. Genome-Wide Association Study Analysis

The GWAS analysis was based on detecting genomic regions of 1.0 Mb that explained more than 1% of the variability in each trait. The percentage of genetic variance explained by the i-th set of SNPs included in a 1.0 Mb window (i-th SNP window) was calculated as described by Wang et al. [45] as

$$\frac{Var(a_{i})}{\sigma_{a}^{2}}\times 100\%=\frac{Var(\sum_{j=1}^{x}Z_{j}{\hat{u}}_{j})}{\sigma_{a}^{2}}\times 100\%$$

where $a_{i}$ is the genetic value of the i-th SNP window of consecutive SNPs; $\sigma_{a}^{2}$ is the total additive genetic variance; $Z_{j}$ is the vector of gene content of the j-th SNP for all individuals and ${\hat{u}}_{j}$ the effect of the j-th SNP within the i-th window.

The GWAS analysis was performed with the POSTGSF90 software v1.83 [46], with the 1.0 Mb overlapping windows option. SNP sets explaining more than 1% of the additive genetic variance were selected. Manhattan plots were generated with R software v4.4.2.

For the ssGBLUP analysis, all animals in the pedigree were included, whereas for the GWAS, only genotyped animals were considered.

### 2.6. Functional Analysis

Potential candidate genes associated within ±1.0 Mb of the significant SNPs were annotated using the Ensembl BioMart database with the latest available cow reference genome (ARS-UCD2.0. https://www.ensembl.org/Bos_taurus/Info/Index (accessed on 20 July 2025). Finally, the functions of these genes and their putative relationships with fertility processes were established by performing an extensive review of the available literature in public databases, as well as in the DAVID V6.8 [47] and UniProt [48] online resources.

## 3. Results and Discussion

In extensive beef cattle systems, the direct selection of females for reproductive traits has been limited, largely because these traits are expressed late and are difficult to measure accurately. However, given the genetic correlations between scrotal circumference (SC) and female fertility [12,49], selecting bulls with higher SC breeding values can improve herd reproductive performance over time [50].

This study represents the first attempt to apply a GWAS joint analysis for male scrotal circumference and cow fertility traits to provide insight into the underlying pleiotropic genetic mechanisms explaining the relationship between scrotal circumference and female fertility traits in the Retinta cattle breed.

### 3.1. Phenotypic Values

Descriptive statistics for scrotal circumference (SC) and female fertility traits are presented in Table 1. The phenotypic values for SC in the Retinta bulls ranged from 25.76 cm to 42.85 cm, with a mean of 33.98 ± 2.27 cm at an average age of 12 months.

Among the 1061 measured bulls, 1005 (95%) exceeded the 30 cm threshold recommended by the Society of Theriogenology [51] and the Beef Improvement Federation [52] for animals ≤ 15 months of age. These measurements also fell within the 30–36 cm range reported by ICAR [53] for bulls of different breeds at one year of age. The mean SC in our study was consistent with previously reported values for the Retinta breed [10,22]. Published SC values for other breeds span a wide range, from 26.4 ± 3.64 cm in Nellore cattle [54,55] to 41.3 ± 3.5 cm in the Rouge des Prés breed (reviewed by Novotná et al. [56]). The overall mean age at first calving (AFC) of Retinta cows was 35.07 ± 5.18 months, similar to the 34.9 ± 7.36 months reported for the Spanish Asturiana de los Valles breed by Gutierrez and Goyache [57]. Published AFC values for other beef cattle breeds range widely, from 23.79 months in Red Angus [58] to 44.13 months in multibreed populations [59]. Average interval between first and second calving (CI12) in the Retinta breed was 15.06 ± 4.01 months. López-Paredes et al. [60] reported CI12 means ranging from 465.4 to 477.6 days in the Blonde d’Aquitaine breed, while Brzáková et al. [61] found a shorter CI12 of 12.51 ± 1.27 months in Charolais and Aberdeen Angus populations. Our mean average calving interval (ACI) was 14.59 ± 2.50 months, lower than the 16.03 ± 5.84 months reported for the Asturiana de los Valles breed [57] and 17.17 ± 8.86 months in Nelore cattle [55]. Reproductive efficiency (RE) exhibited a mean of 75.70 ± 13.55%, similar to the value observed by Jiménez et al. [21] in the same breed (72.05 ± 17.33%). The coefficient of variation was 6.67% for SC, while for cow fertility traits it ranged from 14.77% for AFC to 26.63% for RE, reflecting higher genetic and environmental variability in the Retinta breed and highlighting the potential for improving fertility through selection on these traits.

### 3.2. Heritability Estimates

The estimates of variance components and heritability ($h^{2}$) for SC and female fertility traits are presented in Table 2.

The estimated heritability ($h^{2}$) for the scrotal circumference (SC) was 0.36 ± 0.07, indicating its usefulness as a selection criterion for sires in herds where a genetic response for this trait is desired, such as the Retinta breed. This estimate is higher than the 0.282 ± 0.052 reported by Jiménez et al. [10], which was based on previous BLUP mixed model analyses in Retinta cattle. A wide range of heritability values has been reported, ranging from 0.20 to 0.80 across beef cattle breeds of different countries and types (reviewed by Cammack et al. [6]). The magnitude of $h^{2}$ for SC depends on factors such as population, breed, and the age at which scrotal measurements are taken [8,55,62].

The h^2^ values for female traits in the Retinta breed ranged from low to moderate, suggesting that direct selection on these traits can substantially improve reproductive performance in the Retinta population, considering that these traits generally exhibit low heritability. The $h^{2}$ of AFC in this study was 0.11 ± 0.01, reflecting a low genetic influence. This estimate is lower than those previously reported for the Retinta breed (0.140 ± 0.06, [63]; 0.223 ± 0.046, [10]) and falls within the broader range compiled in the meta-analysis by Cammack et al. [6] for beef cattle. In general, the literature describes AFC heritability as low to moderate. Some studies reported values below 0.1 in 12 American breeds [8,64], while others have found considerably higher estimates, such as 0.235 in the Asturiana de los Valles breed [57], 0.31 in Irish national cattle [65], 0.37 in Panamanian multibreed cattle and Angus [49,59], and 0.27 in the entire population of Charolais and Aberdeen Angus [61]. Our estimated heritability for CI12 was 0.15 ± 0.01, which is higher than those reported in previous studies (0.02 ± 0.004 in Irish national cattle [65]; 0.03 ± 0.01 in Hanwoo cattle [66]), but lower than the values of 0.39 reported by Veselá et al. [67] in the Czech breed and 0.227 reported by Cortes et al. [68] in the Asturiana de los Valles breed.

Heritability estimate for ACI in the present study was 0.19 ± 0.01 and is higher than the values usually reported in the literature. In their review, Koots et al. [69] calculated an average heritability for CI from four published papers of 0.01 and 0.06 for multiparous cows and heifers, respectively. Similarly, $h^{2}$ for CI was 0.125 ± 0.020 in Spanish Asturiana de los Valles breed [57], and 0.105 ± 0.008 in Nellore cattle [55].

Nevertheless, none of these studies have employed RE as an indicator of female fertility. The $h^{2}$ for RE presented a moderate value (0.19 ± 0.01), being similar to the 0.198 ± 0.06 estimated value by Jiménez et al. [10] for RE at last calving, as defined in our study. Also, genetic parameters of RE have been estimated previously in the Retinta breed using repeatability (Rep) and random regression models (RRM) and $h^{2}\;$ estimates were 0.30 ± 0.003 using Rep and ranged from 0.24 to 0.51 with RRM [21]. Similarly, other findings indicate moderate $h^{2}$ values for RE (defined as in our study) in dairy goats [70] and horses [71].

The differences in the magnitude of h^2^ estimates between breeds are influenced by how the trait is defined, the models applied, potential genetic differences, and the effective population size [72].

### 3.3. Genetic Correlations Between SC and Female Fertility Traits

The estimates of phenotypic ($r_{p}$) and genetic correlations ($r_{g}$) between scrotal circumference and female fertility traits are presented in Table 3.

The $r_{p}$ between SC and fertility traits ranged from −0.010 ± 1.014 for SC-ACI to 0.014 ± 0.979 for SC-CI12, while $r_{p}$ within fertility traits ranged from −0.685 ± 3.002 for ACI-RE to 0.582 ± 0.332 for CI12-ACI. In the case of $r_{g}$, although several of them have a low magnitude, considering the standard errors (SE), these correlations would be statistically different from zero. Also, several $r_{g}$ estimates showed relatively high SE. This result is consistent with findings reported by other authors [12,49]. This is likely due to the relatively low number of genotyped females compared to males, combined with the strong environmental component affecting fertility estimates in extensively managed beef cattle, particularly for traits measured throughout the cow’s reproductive life, which generally exceeds 10 years. The $r_{g}\;$ between SC and AFC in this study was negative and favorable, estimated at −0.229 ± 0.114. In a previous study on the Retinta breed, the correlation between this trait and AFC was 0.06, though not statistically significant, and the model used did not include genotypic information [10]. Consistently across the literature, negative correlations between these two traits have been reported since the study by Toelle and Robinson in the Hereford breed [73]. For instance, Bonamy et al. [49], working with Angus herds, found $r_{g}$ values between SC measured at different ages and AFC ranging from −0.478 ± 0.13 to −0.152 ± 0.12. Similarly, Gama et al. [74] observed negative and favorable correlations between SC and AFC from 374 days of age until the final period in Guzera cattle.

Regarding the interval traits, SC showed negative and favorable $r_{g}$ of −0.049 ± 0.121 with CI12 and −0.149 ± 0.110 with ACI. Similar results have been reported for beef cattle. Santana et al. [75] found a negative and favorable correlation of −0.25 between SC and first CI in Nelore cattle. Schmidt et al. [55] also estimated a negative and favorable, although low between SC and CI in the same breed (−0.054 ± 0.038). In line with these findings, Johnston et al. [76] reported $r_{g}$ between SC measured at 6, 12, 18, and 24 months and days to cycling of lactating cows (lactation anestrous interval), ranging from −0.04 to −0.27 in Brahman animals, while in tropical composite cattle, correlations were consistently positive (from 0.13 to 0.23).

In this study, $r_{g}$ between SC and RE was 0.031 ± 0.104. This correlation was previously estimated by Jiménez et al. [21] for SC and RE at different moments in cow lifetime in the Retinta breed and the values were −0.04, −0.091, and −0.15 with RE at 3rd calving, RE at 6th calving and RE at last calving (as defined in the present study), respectively.

The low to very low magnitudes of SC with female reproductive parameters can be explained by the complexity of these traits related to female fertility. These fertility estimators depend on multiple environmental effects (availability of feed on the farm, environmental conditions of temperature and humidity, health status, reproductive management, etc.) that affect the female throughout its whole reproductive life. Environmental effects on fertility are very difficult to control under field conditions, especially in a breed raised extensively. In these cases, the standard approach is to include a contemporary group for comparison, based on herd, year, and season of parity, so that the animals are exposed to the same conditions.

No bibliographical references have been reported on the relationship between SC and RE as defined in our study in other breeds. However, studies have been reported on the genetic correlation between scrotal circumference and several traits related to cow fertility. In Nellore cattle, Van Melis et al. [54] found a positive $r_{g}$ of 0.29 ± 0.05 between SC and heifer pregnancy (the observation that a heifer, exposed to breeding at about 14 months of age, conceives and remains pregnant to palpation) and 0.19 ± 0.05 for SC and stayability (whether a cow gave birth every year up to 5 years old, assuming she had the opportunity to breed). More recently, in this same breed, Schmidt et al. [55] estimated a $r_{g}$ of −0.116 ± 0.020 for SC and gestation length, and −0.084 ± 0.028 for SC and days to calving. Likewise, a strong and positive genetic relationship of 0.76 ± 0.04 was detected by Martinez-Velázquez et al. [8] between SC and stayability (defined as failure or success of a cow in calving at least two calves before six years of age) in the Charolais–Charbray population of Mexico. Conversely, these authors reported genetic correlation close to zero between SC and heifer fertility (defined as failure or success in calving).

Regarding female fertility traits, this study is the first to estimate genetic correlations between them in the Retinta breed. The estimated values ranged from −0.698 ± 0.020 between ACI and RE, to 0.806 ± 0.024 between CI12 and ACI. In other populations, positive correlations between AFC and CI12 have also been reported, including 0.37 in South African Bonsmara cattle [9], and 0.36 in Aberdeen Angus [61]. Moreover, $r_{g}$ between AFC and CI was positive in the studies realized in Irish beef herds [65], Hanwoo cattle [66], and Asturiana de los Valles beef cattle [57], agreeing with the present study. Favorable genetic associations among other reproductive traits have also been observed in other breeds, for example, in Nellore cattle, $r_{g}$ estimates ranged from 0.170 ± 0.040 between gestation length and calving interval, to 0.442 ± 0.050 between days to calving and calving interval [55].

Scrotal circumference is also used as indirect indicator of male fertility. Several studies have demonstrated that SC is associated with libido [77], sexual precocity [78], sperm volume [79,80] and sperm motility [81,82]. In our study on Retinta cattle, SC has been shown to be correlated with quality of semen in bulls, particularly the progressivity (STR and LIN), the longevity (L40 and L50) of the spermatozoa, and the sexual precocity in males [10]. SC is commonly included as a selection criterion in several beef breeding programs worldwide. The Beef Improvement Federation [52] considers scrotal circumference (SC) at one year a key indicator of reproductive efficiency, as it reflects sperm quality and correlates with the age at puberty in both bulls and their daughters. Likewise, ICAR [53] recognizes SC as a reliable measure of sperm production in bulls up to five years old, due to its strong link with testicular size and total sperm output.

### 3.4. Genome-Wide Association Study

**Figure 1:**
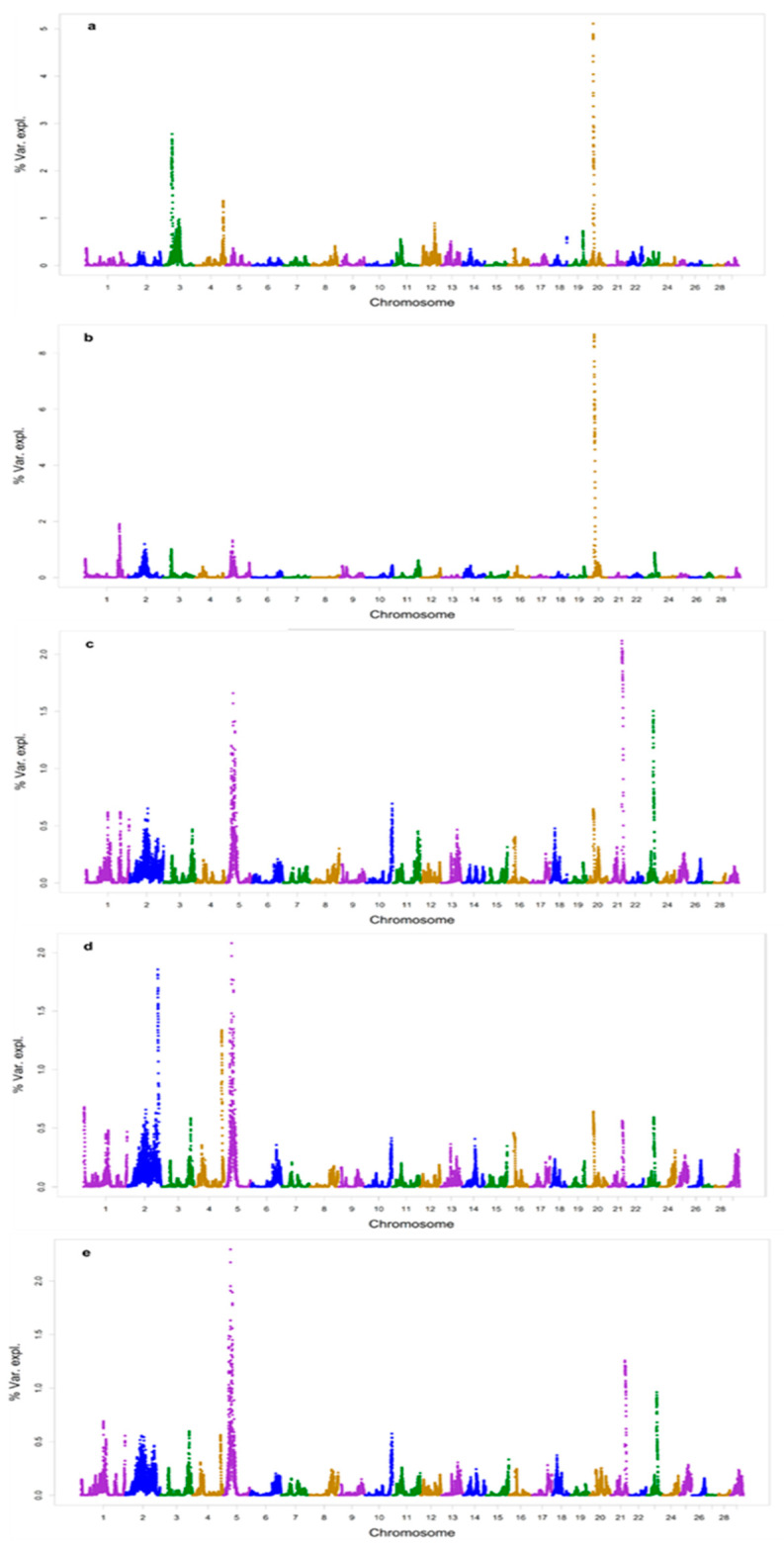
Manhattan plots representing the percentage of explained genetic variance by adjacent SNPs in 1 Mb window for the following traits: (**a**) scrotal circumference (SC); (**b**) age at first calving (AFC); (**c**) interval between first and second calving (CI12); (**d**) average calving interval (ACI); and (**e**) reproductive efficiency at last calving (RE). Chromosomes are represented in different colors.

The ssGWAS revealed several genomic regions containing SNPs for the traits SC, AFC, CI12, ACI, and RE on different chromosomes (Figure 1). The strongest signal for SC was observed on BTA20, followed by BTA3 (Figure 1a). In the case of female reproductive traits, the strongest signal for AFC was observed on BTA20 (Figure 1b), while BTA5 was the chromosome with the highest number of SNPs for both CI12 and ACI (closely followed by BTA2 for CI12) (Figure 1c,d), and the second most prominent one for RE, with BTA21 harboring the most significant signal for the latter (Figure 1e).

In GWAS models based on ssGBLUP, it is common to present the percentage of additive genetic variance explained by the chromosome segment (generally 500 Kb to 1.0 Mb) instead of the level of significance of the association [71,83,84,85,86].

Comprehensive information on the genomic windows identified for SC trait that explained more than 1% of the additive genetic variance, together with the corresponding annotated genes within those regions, is presented in Table 4.

A total of 23 windows were identified. Two of these windows were located on BTA2, with one explaining 1.12% of the variance for the trait, and 22 windows were located on BTA3, including the one that explained the highest proportion of additive variance, 7.53% (Table 4). Within these 23 windows, 198 genomic segments were identified: 118 protein-coding, 58 lncRNAs, 3 miRNAs, 1 rRNAs, 9 snoRNAs, and 9 snRNAs. Some of these genes were associated with male reproduction in cattle and other mammals, supporting the relevance of these regions for fertility.

Genes *HFM1* (Helicase for Meiosis 1) and *MSH4* (MutS Homolog 4) play a role during meiotic recombination, an essential step in the generation of gametes. *MSH4* has a function in the pairing of chromosomes during the early stages of recombination, whereas *HFM1* is involved in the formation of crossovers, thus ensuring a correct chromosome segregation in mid and late stages of this process [87]. Defects in human genes *MSH4* and *HFM1* cause spermatogenesis arrest and azoospermia, leading to male infertility [88,89]. Moreover, Kadri et al. [90] reported variants in both genes associated with abnormal recombination rates in sires. In females, pathogenic variants of the mouse and human genes have been associated with low ovarian reserve, primary ovarian failure, and pregnancy loss [87,91,92,93]. Previous GWAS analysis in Holstein and Aberdeen Angus cattle also support the relationship between these genes and female reproduction [94,95].

Genes involved in defense against oxidative damage also play a key role in fertility. Spermatozoa are especially susceptible to oxidative stress, which is associated with defects in mitochondrial function, motility, viability, capacitation, and fertilizing ability [96,97]. *GSTM3* encodes the most important detoxification enzyme in these cells, and it has been previously associated with reproduction in different species, including cattle. Bulls with lower fertility showed higher levels of protein GSTM3 in sperm, suggesting an increased expression of this gene to compensate for the excessive production of reactive oxygen species (ROS) [82]. Similarly, Dickinson et al. [98] demonstrated that *GSTM3* was upregulated in heifers that conceived early compared with late-conceiving individuals, reinforcing the importance of ROS detoxification in both males and females.

Another biological process of interest in our study is immune response. For example, the colony-stimulating factor 1 (*CSF1*) is present in the testis [99], where it regulates macrophage number and function [100,101]. In the absence of *CSF1*, males exhibit low libido and reduced sperm numbers [102]. In vitro studies have demonstrated that *CSF1* promotes the proliferation of spermatogonia cells in Holstein cattle [103]. This gene is also expressed in the female reproductive tract in several species, e.g., mice, humans, and pigs. In cattle, the temporal and spatial patterns of *CSF1* expression in bovine uteroplacental tissues suggest an important role during pregnancy [104].

It is also worth noticing the presence of gene *SPATA1* (Spermatogenesis Associated 1), whose function is not completely elucidated but has been linked to spermatogenesis. In mice, it is expressed at high levels in testis [105]. The protein encoded by this gene has been found in the acrosomal region of spermatids, where it interacts with IFT20 (Intraflagellar Transport protein 20), which plays a crucial role in spermatogenesis regulation and, thus, fertility [105]. A previous study in cattle demonstrated that the methylation level of *SPATA1* was significantly different in bulls with fertile daughters compared with those with subfertile daughters [106], supporting the implications of *SPATA1* in fertility and the close connection between the reproductive performance of sires and their female offspring.

Other genes reported in our study are involved in different biological processes that are relevant for reproduction, including cell cycle and proliferation, cytoskeleton organization, cell adhesion, gene expression, RNA processing, metabolism, and transport across membranes. The functional annotations of genes associated with SC listed in Table 4 are provided in Table S1 of the Supplementary Materials.

### 3.5. Common Genes Between SC and Female Fertility Traits

The genes located within the windows explaining SC were compared to those found in the windows associated with AFC, CI12, ACI, and RE. Remarkably, our analysis revealed six genes that are common to traits exclusively associated with males (SC) and with females (AFC and CI12), respectively (Table 5). Given their early involvement in reproduction, AFC and CI12 are less affected by environmental and management factors than other traits such as RE and ACI. This could be the reason for the stronger influence of genetics in AFC and CI12 observed in our analysis.

Among the common genes, we found *THSD7B* (thrombospondin type-1 domain-containing protein 7B) on BTA2, which encodes a transmembrane protein that is thought to be involved in protein glycosylation, organization of the actin cytoskeleton, cell adhesion and angiogenesis [48,107]. Not only are these key processes in testicular development and spermatogenesis in bulls, but also in oogenesis, folliculogenesis, and embryo implantation in cows. Expression data show that this gene is expressed in the male and female reproductive systems in cattle and it has been previously associated with fertility in this species and humans [108]. For instance, Madureira et al. [109] found that *THSD7B* was upregulated in the endometrium of highly fertile versus subfertile cows, highlighting its potential role in heifer fertility. The universal functions of *THSD7B* are in line with the implication of this pleiotropic gene in both male- and female-associated parameters observed in this study.

Interestingly, four out of six genes common to traits SC, AFC, and CI12 code for long non-coding RNAs (lncRNAs), pointing to the potential role of these regulatory RNA molecules in both male and female reproduction. lncRNAs are involved in gene expression regulation through different mechanisms. Previous studies have demonstrated the implication of lncRNAs in spermatogenesis, oogenesis, ovarian folliculogenesis, and hormone regulation [91]. For instance, Gm2044 is a mouse lncRNA that is expressed in testis and ovary, where it has a role in sperm development and estrogen biosynthesis, respectively. In males, this lncRNA interacts with different RNA molecules, including gene UTF1 mRNA and miRNA MiR-202, thereby regulating meiosis. Gm2044 also regulates different factors associated with the synthesis of estradiol in granulosa cells [110]. In the same way, the lncRNAs that we found in our analysis (ENSBTAG00000066098, ENSBTAG00000074523, ENSBTAG00000067793, and ENSBTAG00000070030) may interact with other factors involved in fertility, modulating their expression. The next step would be studying their expression levels in tissues of interest and perform a functional validation to confirm their involvement in reproduction. This is an area that remains mostly understudied, especially in livestock. Future research should continue to explore the role of lncRNAs in fertility, with the aim of improving our understanding of the complex interactions underlying this process and, hopefully, use this information to improve reproduction performance.

A previous GWAS performed by Reding et al. [9] in Bonsmara cattle detected several genes associated with SC across different chromosomes, including BTA2 and BTA3. These genes were mainly involved in processes such as development, immunological response, gene expression, and metabolism of carbohydrates and lipids, all of them relevant for reproduction. Although we found different genes associated with this trait, it is also noteworthy that some genes reported in Reding et al. [9] are related to spermatogenesis. For instance, KIF2A (Kinesin-like protein KIF2A) regulates the mitotic divisions undergone by sperm cell precursors during spermatogenesis [111]. Reding et al. [9] also identified genes shared between AFC and CI12; however, no common genes for these traits and SC were reported. Similarly, Fortes et al. [112] conducted a GWAS in Brahman and Tropical Composite sires focused on chromosome X, which included SC, demonstrating that this chromosome, in addition to autosomes, influences the trait.

These results provide a more refined understanding of the molecular architecture of reproduction in extensively managed beef populations and further support SC as a robust selection criterion to enhance both male and female fertility. The integration of genomic data into multi-trait evaluations enables optimized SC selection while accounting for sex-limited traits, thereby minimizing undesirable trade-offs in growth or carcass quality.

This study has some limitations that should be considered. Although several correlations were statistically significant, the genetic and phenotypic associations between SC and female fertility traits were generally low, which may temper the strength of inferences and the predictive value for selection programs. Moreover, the study was conducted in a single, locally adapted breed, Retinta, so caution is needed when extending these findings to other beef cattle populations with different genetic backgrounds or production systems. Female fertility was assessed using indirect measures, such as age at first calving, calving intervals, and reproductive efficiency. While these traits are routinely recorded, they are influenced by both genetic and environmental factors and may not fully capture the underlying biological processes. In addition, the late expression of some fertility traits, combined with practical challenges in collecting data under extensive production systems, can further complicate interpretation. Finally, although the joint GWAS approach offers valuable insights into potential pleiotropic mechanisms, the modest sample size and environmental complexity may have limited the detection of loci with smaller effects.

Overall, this study is the first to apply a joint GWAS analysis to investigate male scrotal circumference and female fertility traits in the Retinta cattle breed. The findings offer new insights into the genetic mechanisms linking these traits, with potential applications for improving breeding strategies in Retinta and other livestock populations.

## 4. Conclusions

Using a multivariate model that included SC and several female fertility traits, pleiotropic molecular markers were identified. Functionally relevant genes, including *HFM1*, *MSH4*, *GSTM3*, *CSF1*, and *SPATA1*, which are involved in biological processes associated with bull fertility such as spermatogenesis, oxidative stress protection, and immune regulation, but are also relevant for reproduction in cows, were highlighted, reflecting the complex regulation of fertility traits. These findings support the use of SC as an indirect indicator of female fertility and, consequently, its application in the Retinta breeding program.

## Figures and Tables

**Table 1:** Summary statistics for scrotal circumference measurement for bulls and female fertility traits in the Retinta cattle breed.

Trait	Mean ± SD	Min	Max	CV (%)
SC (cm)	33.98 ± 2.27	25.76	42.85	6.67
AFC (months)	35.07 ± 5.18	20.15	48.99	14.77
CI12 (months)	15.06 ± 4.01	9.0	24.93	26.63
ACI (months)	14.59 ± 2.50	10.99	24.93	17.16
RE (%)	75.70 ± 13.55	20	100	17.90

SD: standard deviation; CV: coefficient of variation; SC: scrotal circumference; AFC: age at first calving; CI12: interval between first and second calving; ACI: average interval between calving; RE: reproductive efficiency at last calving.

**Table 2:** Variance components and heritability estimates (SE in parentheses) for scrotal circumference and female fertility traits in the Retinta cattle breed.

Trait	$\sigma_{a}^{2}$ (SE)	$\sigma_{cg}^{2}$ (SE)	$\sigma_{e}^{2}$ (SE)	$h^{2}$ (SE)
SC	1.82 (0.35)	1.24 (0.24)	1.98 (0.26)	0.36 (0.07)
AFC	4.80 (0.31)	15.69 (0.36)	22.31 (0.28)	0.11 (0.01)
CI12	2.54 (0.20)	3.07 (0.14)	11.15 (0.19)	0.15 (0.01)
ACI	2.63 (0.15)	2.70 (0.10)	8.38 (0.13)	0.19 (0.01)
RE	50.64 (2.13)	73.17 (1.81)	138.26 (1.74)	0.19 (0.01)

$\sigma_{a}^{2}$: additive genetic variance;$\sigma_{cg}^{2}$: contemporary group variance; $\sigma_{e}^{2}$: residual variance; $h^{2}$: heritability; SE: standard error; SC: scrotal circumference; AFC: age at first calving; CI12: interval between first and second calving; ACI: average interval between calving; RE: reproductive efficiency at last calving.

**Table 3:** Phenotypic (below the diagonal) and genetic (above the diagonal) correlations with standard errors within parentheses between male scrotal circumference and female fertility traits in the Retinta cattle breed.

Trait	SC	AFC	CI12	ACI	RE
SC	-	−0.229 * (0.114)	−0.049 (0.121)	−0.149 (0.110)	0.031 (0.104)
AFC	0.000 (1.001)	-	0.099 (0.052)	0.063 (0.044)	−0.195 * (0.038)
CI12	0.014 (0.979)	−0.005 (1.008)	-	0.806 * (0.024)	−0.410 * (0.036)
ACI	−0.010 (1.014)	0.044 (0.936)	0.582 (0.332)	-	−0.698 * (0.020)
RE	0.009 (0.986)	−0.470 (2.020)	−0.381 (1.755)	−0.685 (3.002)	-

SC: scrotal circumference; AFC: age at first calving; CI12: interval between first and second calving; ACI: average interval between calving; RE: reproductive efficiency at last calving. Asterisk * represents significance level at 5%.

**Table 4:** Annotated genes in the genomic windows within ±1.0 Mb of the significant SNPs that explained more than 1% of the additive variance for SC in the Retinta cattle breed.

ID	Size	Chr	Start	End	Var	Genes
2_60	23	2	60,087,290	61,080,508	1.118	*THSD7B*
3_33	73	3	33,000,242	33,997,602	1.818	*LAMTOR5*, *SLC16A4*, *RBM15*, *KCNC4*, *SLC6A17*, *UBL4B*, *ALX3*, *STRIP1*, *AHCYL1*, *CSF1*, *EPS8L3*, *GSTM3*, *GSTM1*, *GSTM5*, *AMPD2*, *GNAT2*, *GPR61*, *AMIGO1*, *CYB561D1*, *ATXN7L2*, *SYPL2*
3_40	20	3	40,208,518	41,195,205	1.114	*COL11A1*
3_43	18	3	43,619,460	44,610,516	1.594	*PLPPR4*, *PLPPR5*, *U6*, *SNX7*
3_46	16	3	46,086,314	46,922,624	1.515	*DPYD*, *PTBP2*, *U3*, *U6*
3_47	28	3	47,820,211	48,808,060	2.512	*RWDD3*, *TLCD4*, *ALG14*, *CNN3*, *SLC44A3*
3_50	11	3	50,309,627	51,172,850	2.712	*TMED5*, *MTF2*, *DIPK1A*, *SNORA66*, *SNORD21*, *EVI5*, *5S_rRNA*, *GFI1*, *RPAP2*, *GLMN*, *C3H1orf146*
3_51	14	3	51,867,500	52,816,409	2.564	*CDC7*, *HFM1*, *ZNF644*, *BARHL2*
3_53	20	3	53,000,242	53,955,669	2.473	*ZNF326*, *LRRC8D*, *LRRC8C*, *LRRC8B*, *GBP6*
3_54	9	3	54,676,435	55,537,894	1.112	*KYAT3*, *GTF2B*, *PKN2*
3_56	18	3	56,292,899	57,283,356	2.561	*LMO4*, *SNORA62*, *HS2ST1*, *SELENOF*, *SH3GLB1*
3_57	14	3	57,811,390	58,733,254	2.156	*COL24A1*, *ZNHIT6*, *CCN1*
3_58	23	3	58,933,414	59,926,660	4.492	*DDAH1*, *DNAI3*, *MCOLN3*, *MCOLN2*, *LPAR3*, *SSX2IP*, *CTBS*, *SPATA1*, *GNG5*, *RPF1*, *UOX*, *DNASE2B*, *SAMD13*, *PRKACB*
3_60	24	3	60,147,732	61,129,959	6.332	*TTLL7*
3_61	28	3	61,758,600	62,738,616	7.530	*ADGRL2*
3_63	18	3	63,429,032	64,428,546	3.140	*U6*
3_65	29	3	65,163,724	66,162,056	6.773	*ADGRL4*, *IFI44*, *IFI44L*, *bta-mir-10184*
3_66	22	3	66,198,115	67,124,807	1.582	*PTGFR*, *GIPC2*, *U6*, *DNAJB4*, *U6*, *FUBP1*, *NEXN*, *MIGA1*, *U6*, *U6*, *USP33*, *ZZZ3*, *AK5*
3_67	29	3	67,421,386	68,402,961	3.846	*PIGK*, *ST6GALNAC5*, *ST6GALNAC3*
3_68	24	3	68,432,145	69,421,920	1.812	*U1*, *U6*, *ASB17*, *MSH4*, *RABGGTB*, *ACADM*, *SLC44A5*
3_70	20	3	70,167,658	71,141,852	1.612	*ERICH3*, *TNNI3K*, *FPGT*, *LRRIQ3*
3_74	13	3	74,365,922	75,196,565	1.238	*CTH*, *ANKRD13C*, *SRSF11*, *LRRC40*, *LRRC7*
3_76	16	3	76,035,716	76,958,680	1.050	*DEPDC1*, *RPE65*

ID: window identifier; Size: window size in numbers of SNPs; Chr: Chromosome of the window; Start: start position in bp; End: end position in bp; Var: % of explained variance.

**Table 5:** Genes shared across different traits with previous evidence in bovines.

Traits	$V_{SC}$	$V_{AFC}$	$V_{CI12}$	Type	Gene Stable ID
SC, AFC, CI12	1.118	5.478	2.9	protein_coding	ENSBTAG00000021755
				protein_coding	ENSBTAG00000044055 (*THSD7B*)
				lncRNA	ENSBTAG00000066098
				lncRNA	ENSBTAG00000074523
				lncRNA	ENSBTAG00000067793
				lncRNA	ENSBTAG00000070030

$V_{SC}$, $V_{AFC}$, and $V_{CI12}$: additive variance of the windows containing genes that explain scrotal circumference, age at first calving and interval between first and second calving, respectively; Type: type of gene; Gene stable ID: Ensembl identification number for each gene (gene name in parentheses).

## Data Availability

The dataset employed in this study are property of the National Association of Breeders of Selected Retinta Cattle (ACRE) and were provided for scientific purposes under a specific collaboration arrangement. The data set can be made available for scientific purposes to other authors by the ACRE technical department, under reasonable request.

## Supplementary Materials

The following supporting information can be downloaded at https://www.mdpi.com/article/10.3390/vetsci12100977/s1, Figure S1: Manhattan plot for SC; Figure S2: Manhattan plot for AFC; Figure S3: Manhattan plot for CI12; Figure S4: Manhattan plot for ACI; Figure S5: Manhattan plot for RE; Table S1: Functional annotations of genes associated with SC.
