# Supplementary figures and images for: Multivariate Single-Step GWAS Reveals Pleiotropic Genomic Regions and Candidate Genes Associated with Male Scrotal Circumference and Female Fertility Traits in Retinta Beef Cattle
Source: Vet Sci. 2025 Oct 11;12(10):977. doi: 10.3390/vetsci12100977 (PMC12567842; doi:10.3390/vetsci12100977)

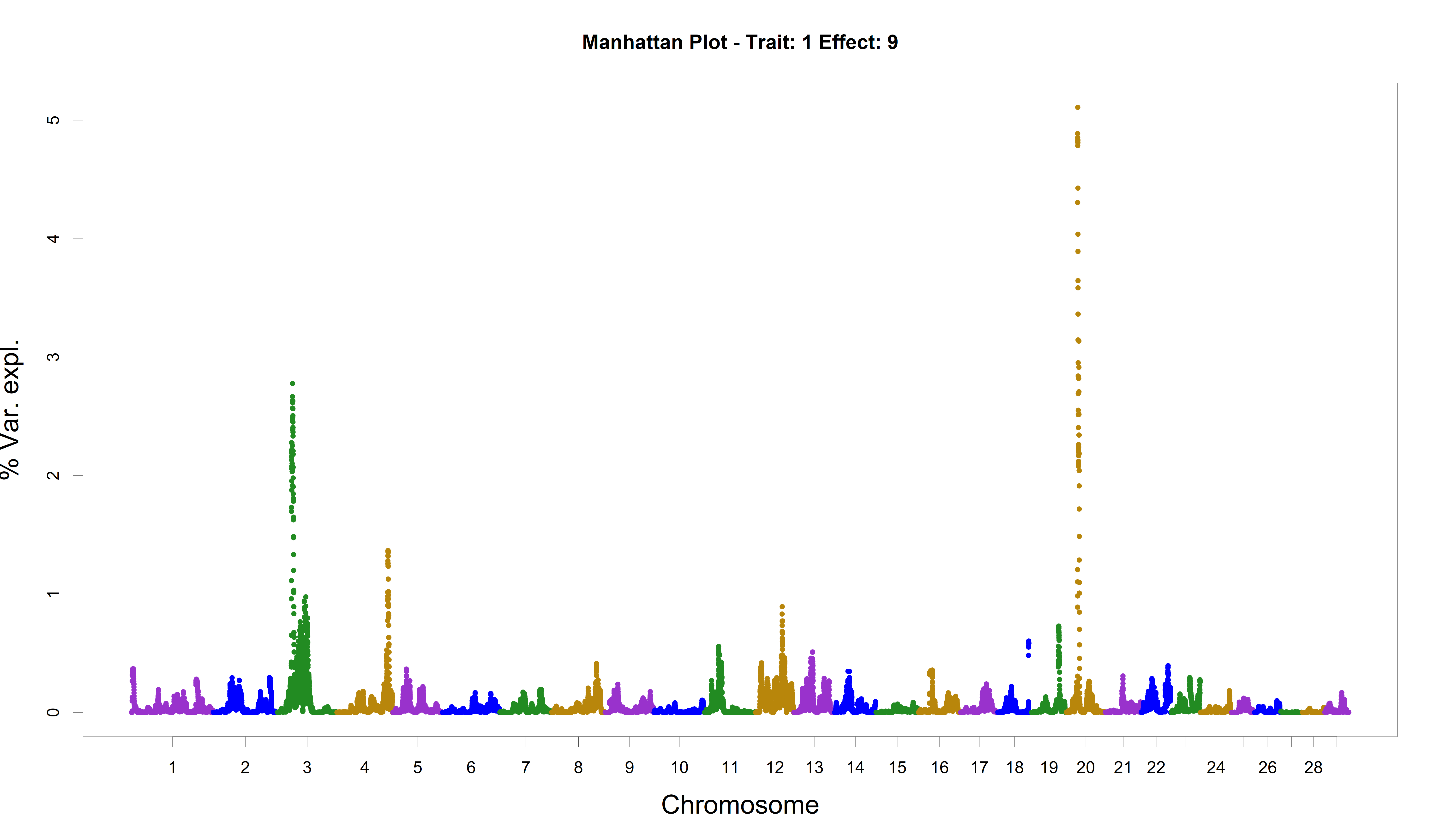

Supplement: Supplementary file 1 [file vetsci-12-00977-s001.zip › vetsci-3885219-supplementary/Supplementary files/Figure S1.png]

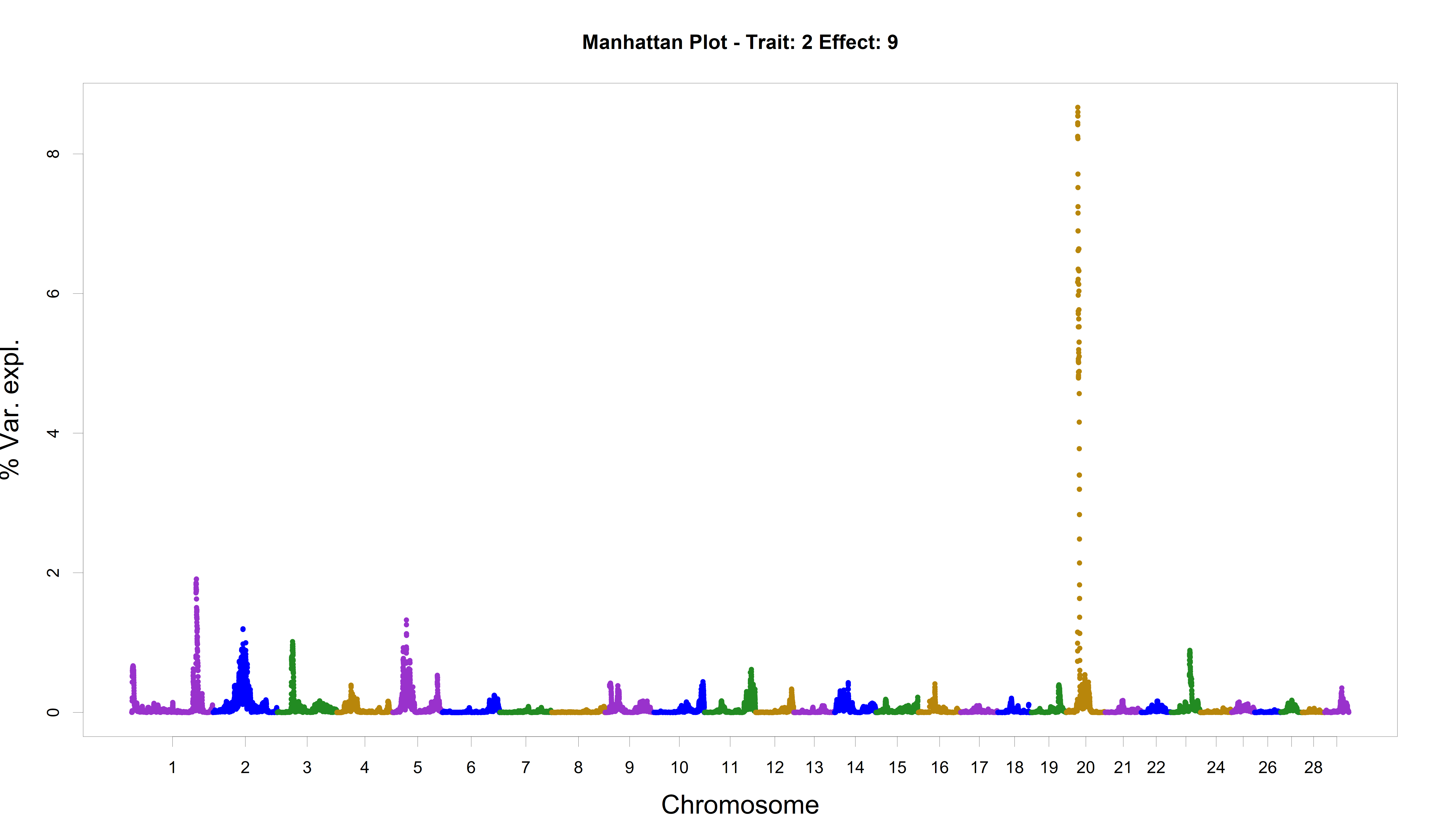

Supplement: Supplementary file 1 [file vetsci-12-00977-s001.zip › vetsci-3885219-supplementary/Supplementary files/Figure S2.png]

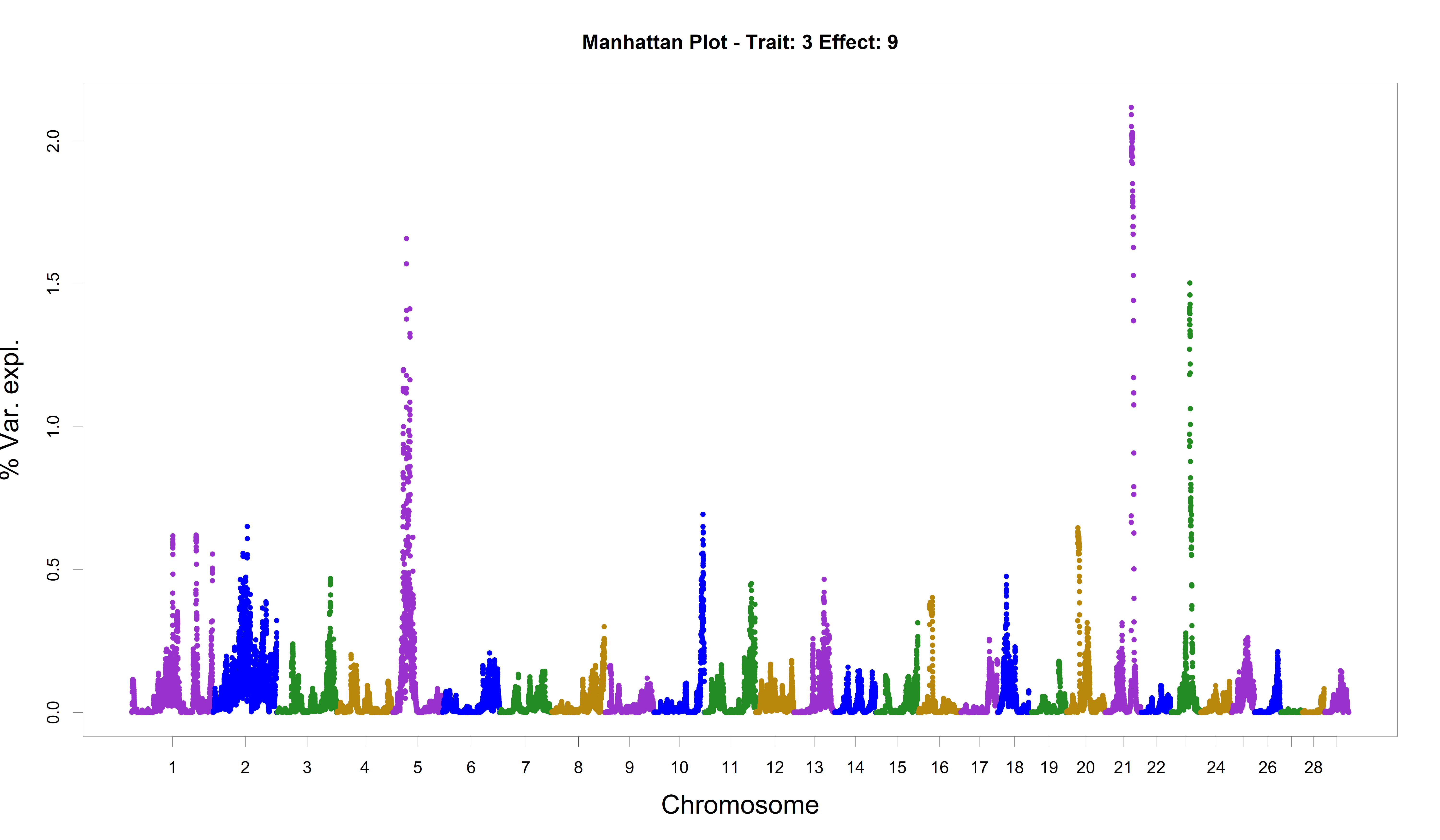

Supplement: Supplementary file 1 [file vetsci-12-00977-s001.zip › vetsci-3885219-supplementary/Supplementary files/Figure S3.png]

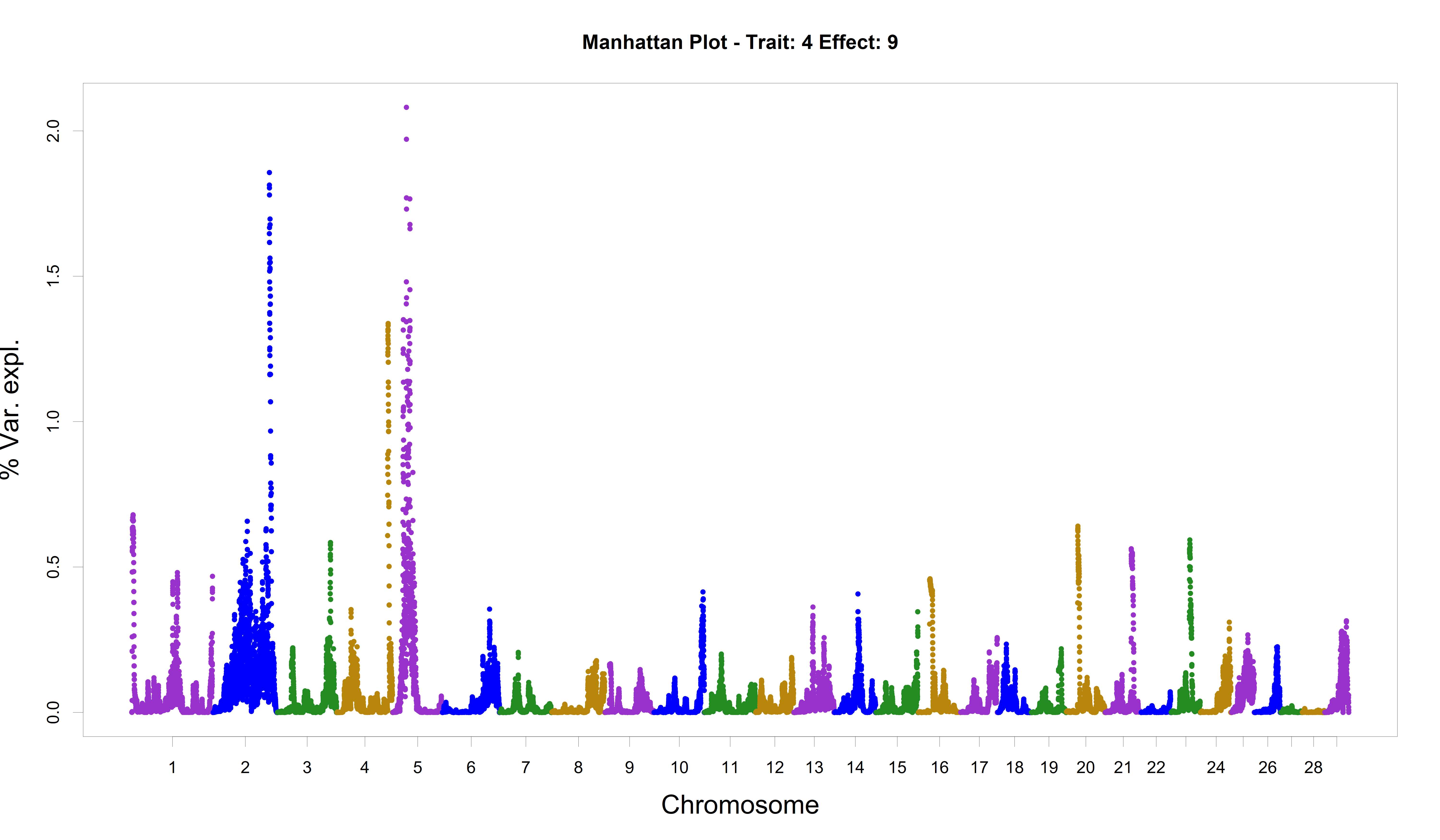

Supplement: Supplementary file 1 [file vetsci-12-00977-s001.zip › vetsci-3885219-supplementary/Supplementary files/Figure S4.png]

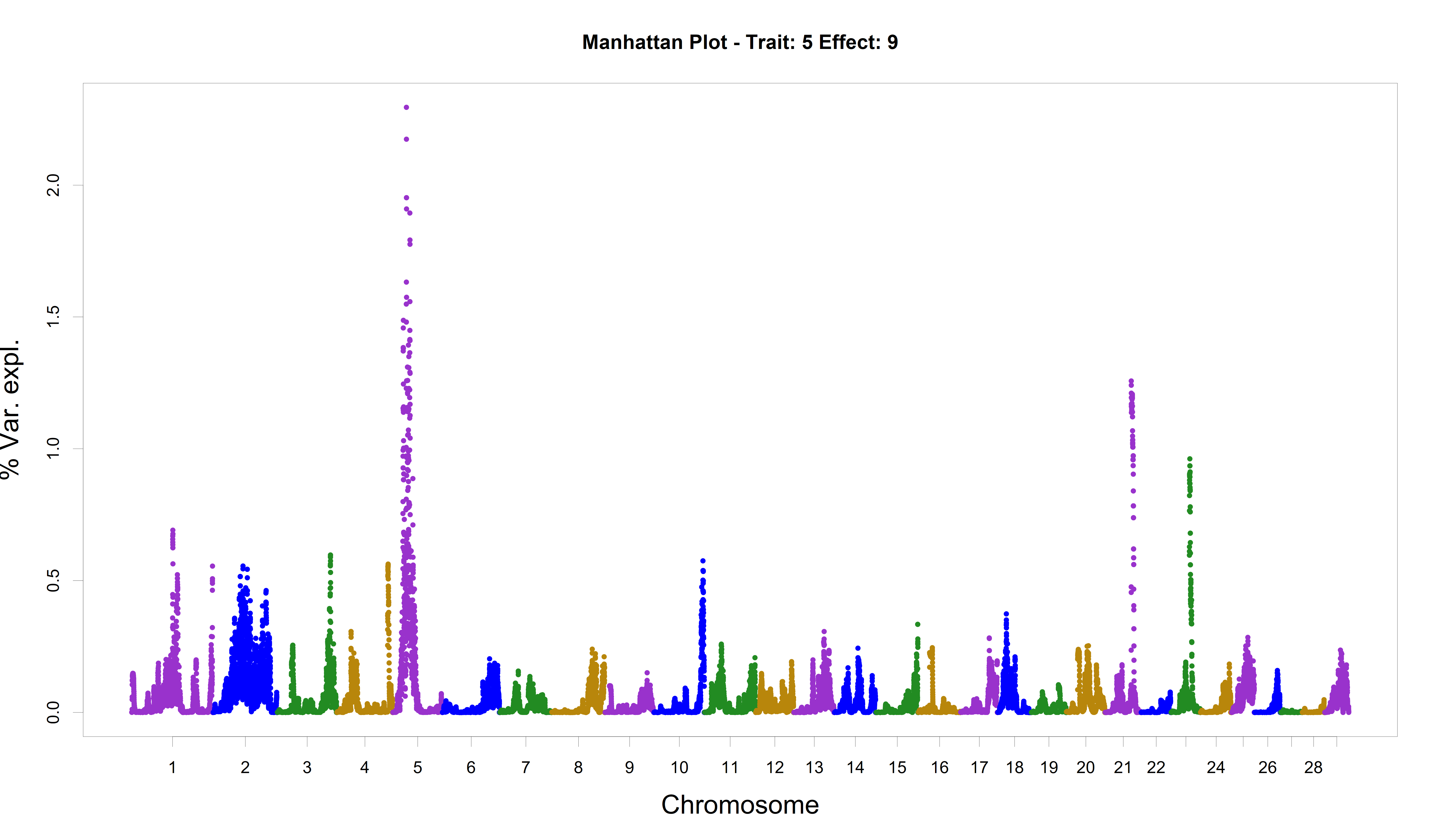

Supplement: Supplementary file 1 [file vetsci-12-00977-s001.zip › vetsci-3885219-supplementary/Supplementary files/Figure S5.png]
